# A 7‐week‐old male Golden Retriever with extreme leukocytosis: A case report

**DOI:** 10.1002/ccr3.3464

**Published:** 2020-11-03

**Authors:** Emily Bingham, Bobbi Conner, Jere Stern, Amber Vitalo, Michael Schaer

**Affiliations:** ^1^ University of Florida College of Veterinary Medicine Gainesville FL USA; ^2^ Virginia‐Maryland Regional College of Veterinary Medicine Blacksburg VA USA

**Keywords:** blood film, cytology, hyperleukocytosis, pediatric

## Abstract

Although neoplasia should be a top concern for extreme leukocytosis in dogs, infectious causes must also be considered to avoid delays in treatment or undue recommendations for humane euthanasia. Blood film review is of paramount importance.

## Graphical abstract

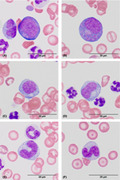

## 1 CASE PRESENTATION

Extreme leukocytosis with leukocytes in excess of 100 × 10^9^/L has not been reported in a veterinary patient with an identified bacterial source and is commonly associated with neoplastic processes. Blood film review was crucial in excluding the more common hematologic malignancies and allowed for alternative differentials with improved prognoses.

A 7‐week‐old, male, intact Golden Retriever was presented with a history of failure to thrive manifesting as stunted growth in comparison to littermates despite adequate nutritional intake. Two days prior to presentation the patient was treated with an unknown dewormer after a fecal sample tested positive for hookworms. At that time, he was diagnosed with a grade 4/6 heart murmur and had an increased respiratory effort. He was referred to the University of Florida Small Animal Hospital Emergency & Critical Care service for further evaluation and possible diagnostics. On presentation, abnormal physical examination findings included a grade 4/6 bilateral apical systolic heart murmur, bounding femoral pulses, an increased respiratory effort, dull mentation, and bruising in his axilla and inguinal areas. Initial vital signs included a temperature of 101.8°F (38.8°C), heart rate of 100 beats per minute, and he was panting.

**FIGURE 1:**
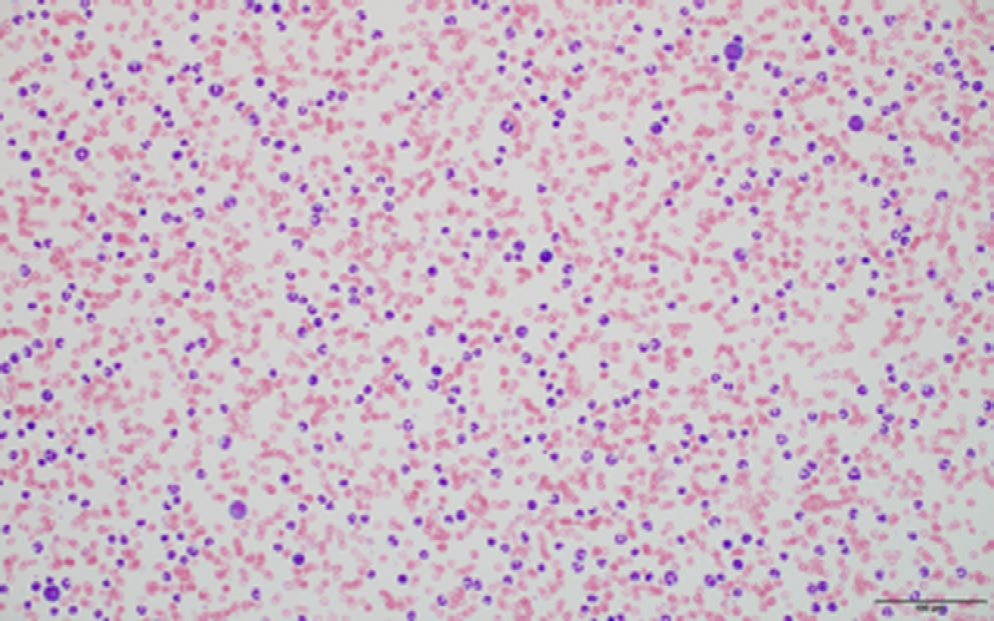
Low power (20×) photomicrograph of blood film from a dog with extreme leukocytosis

**FIGURE 2:**
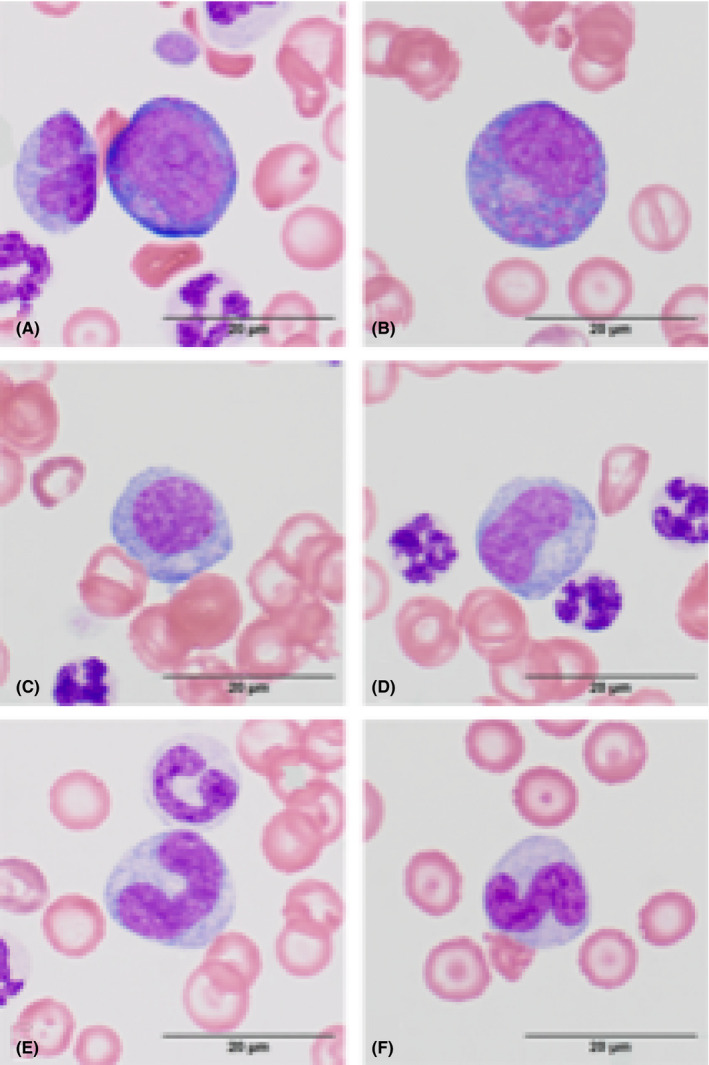
Photomicrographs of leukocytes seen in a blood film from a dog. H&E stain; bar = 20 µm. A, Presumed myeloblast with two visible nucleoli. The presence of eosinophilic cytoplasmic granules indicates this cell may be a late‐stage myeloblast. B, Promyelocyte with many eosinophilic primary granules within the cytoplasm and no visible nucleolus. C, Myelocyte with a round nucleus and no visible primary granules. D, Metamyelocyte with a reniform nucleus. E, Giant toxic band (bottom) next to a toxic neutrophil (top). Giant bands are indicative of marked toxic changes secondary to inflammation. F, S‐shaped band with toxic cytoplasm (cytoplasmic basophilia)

A CBC (Procyte Dx; IDEXX Laboratories) revealed a severe normocytic, normochromic anemia (hematocrit [Hct] 0.118 L/L, reference interval [RI] 0.27‐0.37; mean cell volume [MCV] 65.9 fL, RI 63‐74; mean cell hemoglobin content [MCHC] 305 g/L, RI 295‐341), with mild anisocytosis, polychromasia, and hypochromasia all observed on later blood film evaluation.^1^ There was a mild thrombocytopenia (109 × 10^9^/L, RI 193‐653), with no clumps noted on later blood film evaluation, as well as an extreme leukocytosis (165.37 × 10^9^/L, RI 8.8‐22.4).^1^ A blood film made at time of collection (Figures 1 and 2) was submitted for evaluation and manual leukocyte differential by a clinical pathologist the following day, which revealed an extreme neutrophilia with a regenerative left shift (segmented neutrophils 110.8 × 10^9^/L, RI 4.1‐12.2; banded neutrophils 31.4 × 10^9^/L, RI < 0.3; metamyelocytes 5.0 × 10^9^/L; myelocytes 1.7 × 10^9^/L) and moderate monocytosis (8.3 × 10^9^/L, RI 0.5‐1.6).^1^ Toxic changes were very prominent, with most neutrophils having cytoplasmic Döhle bodies and basophilic cytoplasm, along with occasional cytoplasmic vacuolization and infrequent giant bands and metamyelocytes (Figure 2). Presumed myeloblasts and promyelocytes were present (Figure 2), though not in sufficient numbers to be included in the leukocyte differential. No significant granulocyte dysplastic changes, excluding giant bands and metamyelocytes, were observed. Very rare rod‐shaped structures, consistent with bacterial bacilli, were observed intracellularly and extracellularly, raising strong concern for bacterial septicemia. A serum biochemistry panel revealed a moderate increase in alanine phosphatase (388 U/L, RI 93‐221) and γ‐glutamyl transferase activity (16 U/L, RI 0‐2); and mildly decreased urea nitrogen (0.2 mmol/L, RI 2.5‐6.2), creatinine (0.1 μmol/L, RI 35‐61), and albumin (20 g/L, RI 20‐31).^1^ A urinalysis was unremarkable other than evidence of isosthenuria (urine specific gravity [USG] = 1.012) and 2 + bilirubinuria (Multistix 10 SG; Siemens Healthcare). PCV and total solids were 17% and 60 g/L, respectively, with clear serum, blood glucose was 137 mg/dL on glucometer, and Doppler‐determined systolic arterial blood pressure was 160 mm Hg. Echocardiography was performed by a cardiologist and revealed trace to mild mitral and tricuspid regurgitation and mildly enlarged left ventricle and left atrium. A point of care ultrasound of the abdomen was performed that showed no peritoneal effusion and aerobic and anaerobic blood cultures were submitted. The dog was hospitalized overnight and treated with a packed red blood cell transfusion (10 mL/kg [4.5 mL/lb], IV) and ampicillin/sulbactam (30 mg/kg [13.6 mg/lb], IV, q 8 hours).

The patient's mentation and physical activity were mildly improved on the second day, and he was eating and drinking well. The post‐transfusion PCV was 21% and TS was 54 g/L with clear serum. An abdominal ultrasound examination was performed by a radiologist, and the findings included numerous hepatic nodules, some with a cavitated fluid‐filled central portion without evidence of blood flow; diffusely mottled/micronodular splenomegaly; renal perihilar steatitis; diffuse gastrointestinal fluid with minimal motility; scant, mildly echogenic peritoneal effusion; and mesenteric lymphadenopathy. A sample of effusion was collected and submitted for aerobic and anaerobic culture and susceptibility. No additional tests were performed due to financial restrictions. Due to these findings, enrofloxacin (10 mg/kg [4.5 mg/lb], IV, q 24 hours) was added to his daily therapy.

On the third day of hospitalization, the puppy remained static in attitude and appearance. A fecal floatation was performed with no ova seen. A sample for genotype testing was submitted to screen for CLAD (Optigen LLC). Due to financial constraints, the patient was discharged with the owner to provide continued monitoring and care at home. Amoxicillin/clavulanic acid (13.75 mg/kg [6.25 mg/lb], PO, q 12 hours) and enrofloxacin (5 mg/kg [2.2 mg/lb], PO, q 24 hours) were to be continued at the same doses for the next 28 days.

Three days after discharge, the patient returned for a scheduled recheck. The owners reported he had been doing well at home; he was energetic and playing with his littermates as well as eating and drinking. The physical examination findings were completely normal and the previously auscultated heart murmur was no longer heard. The CBC (Advia 2120i Hematology System; Siemens Healthcare) revealed a persistent, though markedly improved, leukocytosis (31.21 × 10^9^/L, RI 8.8‐22.4), moderate macrocytic hypochromic anemia (Hct 0.249 L/L, RI 0.27‐0.37; MCV 77.1 fL, RI 63.0‐74.0; MCHC 292 g/L, 295‐341), neutrophilia (18.7 × 10^9^/L, RI 4.1‐12.2) with a regenerative left shift (banded neutrophils 3.1 × 10^9^/L, RI < 0.3; metamyelocytes 0.31 × 10^9^/L), and monocytosis (4.1 × 10^9^/L, RI 0.5‐1.6).^1^ Results from the blood culture returned *Staphylococcus pseudintermedius* as well as *Clostridium bifermentans*; the ascites fluid cultured the *Staphylococcus pseudintermedius* with no growth in the anaerobic sample. Based on susceptibility results his antibiotic therapy was changed to amoxicillin/clavulanic acid alone.

The final recheck occurred 18 days after initial presentation. The puppy was continuing to thrive, no murmurs or arrhythmias were ausculted, and he was tolerating his medication well. A repeat CBC (Advia 2120i Hematology System; Siemens Healthcare) showed continued improvement, with total leukocyte and neutrophil counts within reference intervals (WBC 11.19 × 10^9^/L, RI 8.8‐22.4; neutrophil 7.7 × 10^9^/L, RI 4.1‐12.2) without evidence of anemia (Hct 0.288 L/L, RI 0.27‐0.37).^1^ The CLAD genetic testing showed no mutation.

The patient was ultimately adopted to another family (the original owner was also the breeder) in a distant geographic location, precluding further follow‐up at the University of Florida. About 12 months after the original presentation, a follow‐up telephone call to the new owners revealed a description of a normal, apparently healthy, and active Golden Retriever dog.

## 2 DISCUSSION

The severe leukocytosis in this dog initially raised suspicion for some type of neoplastic process as the cause of this patient's illness, particularly owing to his lack of severe systemic illness. White blood cell counts above 100 × 10^9^/L in dogs are most commonly associated with hemic neoplasms, paraneoplastic responses, leukocyte adhesion deficiencies, granulocyte‐colony stimulating factor administration, and infection with *Hepatozoon americanum*.^2^ CLAD is a primary immunodeficiency, described in Irish setters and Irish Setter crosses, caused by a lack of the β‐2 integrin subunit (CD18) that can result in extreme leukocytosis due to leukocytes’ inability to extravasate from the vessels.^3^ While it was considered unlikely such prominent and profound left shifting could be due to CLAD alone, the combination of both increased leukocyte production due to inflammation and decreased trafficking to tissues could be the cause of such an extreme leukocytosis. Although *Hepatozoon americanum* has been reported in Florida, it is not commonly reported in the specific region where this puppy was seen and no relevant travel history was reported. In retrospect, this should have remained an important differential in this patient; however, the dog's improvement with antimicrobial treatment and the financial limitations of the client precluded further diagnostic investigation.

As this dog was initially presented to the hospital outside of the usual business hours, a review of the blood film by a clinical pathologist was not performed until the following morning. The slide review was essential for identification of a putative extreme inflammatory response rather than a leukemia. Non‐neoplastic, infectious hyperleukocytosis, defined as a white blood cell count >100 × 10^9^/L, is infrequently reported in the human medical literature and has rarely been reported in the veterinary literature, excluding *H americanum*.^4^ Moreover, most of veterinary research defines a white blood cell count of >50 × 10^9^/L as “an extreme leukocytosis,” and as an “extreme neutrophilic leukocytosis” when there is a white blood cell count >50 × 10^9^/L of which more than 50% are neutrophils.^5^, ^6^ The human medical literature attributes most causes of hyperleukocytosis to neoplastic diseases; however, there are reports of it being caused by infectious agents, particularly in infants. It is interesting that in people the few cases of hyperleukocytosis not caused by cancer are seen in very young patients,^7^ as this was also true for the dog in this report.

In a retrospective case series of 104 cats with neutrophilic leukocytosis, the authors set inclusion criteria guidelines for patients to have a leukocytosis >50 × 10^9^/L with >50% neutrophils. That study found that 37% of cases were caused by infectious diseases, 23% by neoplastic diseases, 22% by immune‐mediated diseases, and 18% by tissue necrosis.^5^ They did not report a differentiation of disease process related to severity of the leukocytosis. This particular report indicated a mortality rate of 61%, consistent with the mortality rate for dogs (62%), but higher than what is reported for humans (31%).^5^, ^8^ This same study concluded that this high mortality should be carefully considered when determining prognosis and outcome.^5^ A more recent retrospective case study involved 361 dogs with “extreme leukocytosis” defined as a total white blood cell count ≥35 × 10^9^/L. This paper concluded that the majority of the “extreme leukocytosis” was caused by bacterial or fungal infections, babesiosis, immune‐mediated hematologic disease, and tissue necrosis; and that a “marked leukocytosis” was more likely to indicate serious disease in dogs.^9^ Again, this study did not specifically correlate severity of the leukocytosis with diagnosis; however, they did report three dogs with hemic neoplasms, all with WBC counts > 100 × 10^9^/L; they further defined degree of leukocytosis in dogs with babesiosis, and all dogs in this subgroup had WBC counts < 100 × 10^9^/L.^9^ Reports of dogs with WBC counts > 100 × 10^9^/L with non‐neoplastic causes are rare in the veterinary literature, with the notable exception of dogs diagnosed with *Hepatozoon americanum*.^4^

Decreased concentration of urine has been considered normal in puppies <8 weeks of age due to immature renal function, but our patient had a USG that was lower than the normal reference range for his age group (USG 1.012, RI 1.015‐1.044).^10^ A range of variability is expected in healthy dogs, and because we only have one reported USG we cannot definitively conclude that our patient had a renal insufficiency resulting in isosthenuria; however, hepatic injury could explain both his isosthenuria and 2 + bilirubinuria. When these findings are reviewed with the abnormal serum biochemistry findings (alanine phosphatase 388 U/L, RI 93‐221; γ‐glutamyl transferase activity 16 U/L, RI 0‐2; urea nitrogen 0.2 mmol/L, RI 2.5‐6.2; creatinine 0.1 μmol/L, RI 35‐61; albumin 20 g/L, RI 20‐31) and abdominal ultrasound findings, one could consider a degree of cholestasis and hepatic injury. The bacteremic source for the hepatic injury, thought to be secondary to either hepatic abscesses or areas of malignancy, was not identified.

Our case report has some significant limitations, including differences in the laboratory analysis performed throughout the case and financial constraints restricting hospitalization and diagnostic testing. Because the patient initially was presented outside of normal business hours, his initial CBC was performed on a different machine than the subsequent CBCs. Further, the hematocrit on the initial CBC was quite different from the spun PCV (11.8% vs. 17%, respectively). The Hct is a calculated value that relies on the MCV and the red blood cell count being measured accurately by an automated hematology instrument with no complicating factors whereas the PCV is reliant on manual interpretation. An explanation for this discrepancy is not readily apparent, but could have been due to machine error or pre‐analytical error.

Our top considerations for the cavitated hepatic nodules included hepatic abscesses or areas of malignancy, so a cytologic review of the abnormalities in addition to the performed diagnostics would have further aided our clinical diagnosis and therapeutic plan. Due to financial limitations, we did not obtain samples of the liver or the cavitated hepatic nodules for culture or bone marrow aspirate for cytologic evaluation, nor was a repeat abdominal ultrasound performed during any of the recheck examinations, precluding identification of the source of bacteremia in our patient. Despite this, the experience underscored the importance of blood film review. Neoplasia is the most likely differential for a patient presenting with extreme leukocytosis and was our initial concern. It was not until the hemogram slide report was available that bacteremia became our primary differential and focus of therapy. This information dramatically changed the prognosis presented to the owner, altering the outcome for the patient. This case report highlights the importance that although neoplasia is still an important differential in patients with hyperleukocytosis a clinician blood film review would identify the presence of left shifting and toxic changes resulting in additional differentials that carry potentially better long‐term prognoses. Although the term hyperleukocytosis is not commonly used in veterinary medicine, it is reported in the human medical literature. We found the term beneficial for use during our literature searches as we sought assistance during the management of the patient presented in this case report.

## CONFLICT OF INTEREST

The authors have no conflicts of interest to disclose.

## AUTHOR CONTRIBUTIONS

EB, BC, and AV: managed the patient. EB, BC, JS, and MS: wrote the manuscript. All authors reviewed and approved the final version of the manuscript.

## Data Availability

The data that support the findings of this study are available from the corresponding author upon reasonable request.
